# Linking hypotheses underlying Class A and Class B methods

**DOI:** 10.1017/S095252381300045X

**Published:** 2013-11

**Authors:** M.J. MORGAN, D. MELMOTH, J.A. SOLOMON

**Affiliations:** 1 Max-Planck Institute for Neurological Research, Cologne, Germany; 2 Division of Optometry and Visual Science, City University London, London, UK

**Keywords:** Psychophysics, Linking hypotheses, 2AFC, Method of single stimuli

## Abstract

Class A psychophysical observations are based on the linking hypothesis that perceptually distinguishable stimuli must correspond to different brain events. Class B observations are related to the appearance of stimuli not their discriminability. There is no clear linking hypothesis underlying Class B observations, but they are necessary for studying the effects of context on appearance, including a large class of phenomena known as “illusions.” Class B observations are necessarily measures of observer bias (Fechner’s “constant error”) as opposed to Class A measures of sensitivity (Fechner’s “variable error”). It is therefore important that Class B observations distinguish between response biases, decisional biases, and perceptual biases. This review argues that the commonly used method of single stimuli fails to do this, and that multiple-alternative forced choice (mAFC) methods can do a better job, particularly if combined with a roving pedestal.

## Introduction

Despite being called a materialist by none other than Vladimir Ilyich Lenin, Thomas Henry Huxley was a subtle dualist. Quoting a notorious remark by the French physiologist, he objected: “When Cabanis said that thought was a function of the brain, in the same way that bile secretion is a FUNCTION of the liver, he blundered philosophically. Bile is a product of the transformation of material energy. But in the mathematical sense of the ‘function’ thought may be a function of the brain. That is to say, it may arise only when certain physical particles take on a certain order.” (Letter from T.H. Huxley to Mr. McClur, 1881; original emphasis.)

For a relation from domain *X* to range *Y* to be considered a function, distinct values in *Y* must correspond to distinct elements in *X*. The square root can thus be considered a function, but the arctangent cannot because distinct values in its range correspond to the same element in its domain (e.g., arctan 0 = 0 and arctan 0 = *π*). It will be seen that Huxley’s idea of a functional relation from brain states to perception implies that two different perceptions must correspond to two different brain states. Nearly all neuroscientists believe that Huxley’s idea is correct, but they would also concede that it would not be testable for many thousands, perhaps millions of years, because we cannot state with confidence when two brain states are identical. Time travel would be a useful tool for doing this but is not part of the neuroscientist’s toolkit.

Brindley (1960, p. 144) proposed what was intended to be a useable version of Huxley’s idea and called it a “linking hypothesis”: “…whenever two stimuli cause physically indistinguishable signals to be sent from the sense organs to the brain, the sensations produced by these stimuli, as reported by the subject in words, symbols or actions, must also be indistinguishable” (Brindley, 1960, p. 144). This is saying that two different sensations cannot correspond to a single brain state, in other words that sensations are a function of brain states. An example of Brindley’s linking hypothesis is that if two lights cause identical quantum catches in the three classes of cone, then they must cause the same sensation. The linking hypothesis does not state that two lights giving rise to the same sensation of color must cause identical quantum catches nor should it (consider the case of central achromatopsia). Therefore, we cannot directly infer anything about brain states from the case of equal sensations. A more useful version of the linking hypothesis is that if two sensory inputs to the brain cause different sensations, then they must have different effects on the brain. In other words, if we can discriminate between two stimuli, there must be at least one neuron that can also discriminate them. The task of the neuroscientist is to find the locus of these neurons. As Teller and Pugh put it:

Most visual scientists probably believe that there exists a set of neurons with visual system input, whose activities form the immediate substrate of visual perception. We single out this one particular neural stage with the name: the *bridge locus*. The occurrence of a particular activity pattern in these bridge locus neurons is necessary for the occurrence of a particular perceptual state; neural activity elsewhere in the visual system is not necessary…. Most visual scientists would agree that they [*sc* the bridge neurons] are certainly not in the retina (Teller & Pugh, 1983, p. 581, parenthesis added).^1^

Examples of this approach are the measurement of neurometric functions corresponding to the psychometric functions for vernier acuity (Parker & Hawken, 1985; Hawken & Parker, 1987) and motion direction discrimination (Newsome et al., 1989).

No meaning can be given to the statement that two inputs give rise to the “same” sensation unless we have an established method for determining that they are not different. To show that two lights are metameric requires a demonstration that the observer required to discriminate between them performs at chance, which in practice means not significantly better than chance. Conversely, the fact that two lights are not metameric must entail that discrimination performance is significantly greater than chance. For a shortcut that avoids measuring probabilities, the method of adjustment is often used, which allows the observer to minimize the perceived difference between two stimuli. The fact that this method works at all deserves more comment than it usually gets. It must mean that observers can set a criterion for determining when two stimuli are as similar as possible, else they would continue their adjustment for ever. One has only to imagine trying to get a rat to set a metameric match (without reinforcement, evidently) to see how interesting it is that humans can perform the task reliably. We accept the method of adjustment only because it is logically underpinned by the measurement of probabilities.

Our ability to measure discrimination by probabilities without a subjective element (defined below) is what makes psychophysics a branch of applied physics and explains the emphasis Brindley placed upon what he called “Class A” observations. Class A observations define the class of stimuli that are discriminable, and by exclusion, those that are indiscriminable. Class B observations are all the rest. W.S. Stiles, a physicist working at the UK National Physical Laboratory at Teddington, carried out only Class A observations and would decline invitations to comment on how many unique hues there were, or whether there is a sharp divide between the sensations of blue and green (Mollon, 1986). The aim of Class A observations is to take the psyche out of psychophysics. The fact that the just noticeable difference (JND) in vernier offset is in the hyperacuity range of ∼5 arcsec is a Class A observation. The fact that two vertically aligned Gabor patches with stationary envelopes appear misaligned if their carrier gratings drift in opposite directions is a Class B observation (De Valois & De Valois, 1991). This example illustrates, as Brindley emphasized, that Class B observations are not at all uninteresting, but they do have a different logical status from Class A because they lack the clarity of a clear linking hypothesis.

Another kind of distinction, related to Class A *versus* Class B, is explained by Sperling et al. (1990). A Type 1 observation gives an observer a task to perform that has a correct answer, such as whether one grating has a higher contrast than another or whether a line is tilted clockwise or anticlockwise of the vertical. The observer could be trained to perform the task without any verbal instructions if given feedback (aka “reinforcement”). Sperling et al.’s criterion for a Type 1 task, therefore, relates to another, the “pigeon test” (John Mollon, personal communication). If you can train a pigeon to perform the task, it is Class A/Type 1. If not, it is Class B. All “illusions” are Type B. This is why it is difficult to demonstrate them in animals. A pigeon could be trained to peck at the longer of two lines with reinforcement and then given a transfer test without reinforcement to the two versions of the Muller-Lyer figure. If it pecked the outgoing arrowhead version, we would have learned very little. The bird might have learned in the original training to peck the figure with the larger outline. There is no transfer test that can ever show that the bird “sees” the line with the outgoing arrowheads as longer.

However, the criterion of a task having a correct answer is only necessary for it to be Class A, not sufficient. Consider again the illusion of the misaligned, drifting gratings within stationary envelopes. There is a correct answer in this task, namely that the envelopes are aligned, but it is not the answer we want the observer to give, so we are unable to use feedback/reinforcement.^2^ The explanation of this apparent paradox is that if we measure a psychometric function, using a question to which there is a correct answer, there are two things that we can measure: the slope of the function, which is a measure of discrimination, and the central tendency of the function, which is a bias. The underlying distinction between Class A and Class B observations is not whether there is a correct answer or whether we use the method of “forced choice,” but whether we measure the JND or the bias. Some investigators appear to think that they can avoid biases by using a method of forced choice, but there they “blunder philosophically.”

Signal detection theory clarifies the distinction between discriminability and bias with the twin concepts of internal noise and the observer’s criterion. Discriminability is limited by internal noise, and the aim of a bias-free method is to measure that internal noise. The decisional criterion is a rule that the observer uses to select a response, given the sensory data. For example, in making a vernier acuity decision with the method of single stimuli (MSS), the observer has access to a noisy signal with a value along a dimension going from “left” to “right” but must select some value along that criterion to define as the watershed. If there is an illusory offset (De Valois & De Valois, 1991), we find that the midpoint of the psychometric function has shifted from zero. This could be because the position of the signal + noise function has shifted along the sensory continuum or because the position of the decisional criterion has changed. From the point of view of signal detection theory, these alternatives are logically the same, and this is the crux of the difficulty with Class B observations.

To make this point, Morgan et al. (2012) attempted to see whether observers could voluntarily shift their bias with the MSS without affecting the slope of the psychometric function. An increasing number of reports in visual cognition seem to be measuring perceptual biases due to adaptation, attention, and illusions with the MSS, without ruling out the possibility that these biases are due to changes in the observer’s decisional criterion. Examples include: (i) a movement aftereffect caused by imagining the adapting stimulus (Winawer et al., 2010), (ii) a decrease in the motion aftereffect caused by distracting attention from the adaptor (Taya et al., 2009) and the apparently spatiotopic version of the tilt aftereffect due to motion (Turi & Burr, 2012).

**Fig. 1.**
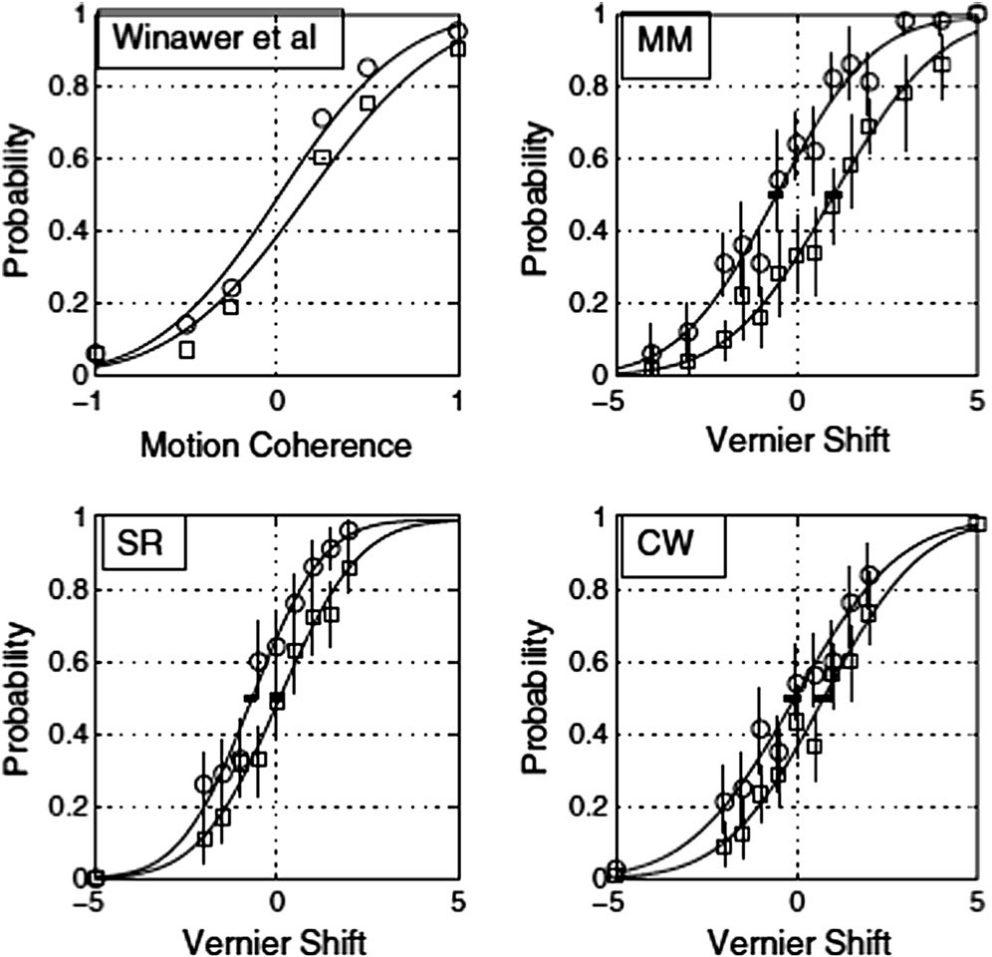
The first panel (top left) shows data from Winawer et al. (2010). The vertical axis shows the probability of classifying the motion direction left *versus* right. The circles show data from the unadapted condition, the squares show the effect of adapting to an imaginary moving stimulus. The remaining panels show psychometric functions from a three-dot vernier alignment task in which the magnitude of the physical shift of the center dot (horizontal axis) was sampled from a set of fixed values without replacement. The units of displacement are Weber fractions as percentages (100 × target shift/interpatch distance). Negative shifts are shifts “down.” The vertical axis is the probability with which the observer classifies a shift as “up” *versus* “down.” Vertical bars are 95% confidence limits based on the binomial distribution. The circles show data taken with the observers’ natural biases and the rectangles with a deliberately feigned bias in the opposite direction. All curves are best-fitting two-parameter (*μ*, *σ*) cumulative Gaussian functions. The small horizontal bars at 0.5 on the ordinate show the 95% confidence intervals for the mean of the psychometric function *μ*, obtained from 160 simulated runs of the experiment using the maximum-likelihood fits of *μ* and *σ*.

Fig. 1(top left) shows data replotted from Winawer et al. (2010), showing the typical horizontal shift of the psychometric function due to adaptation in the MSS. As is commonly found, the shift is less than the standard deviation of the psychometric function and is thus less than the conventionally defined JND. The other panels in Fig. 1 shows results for a three-stimulus vernier alignment task in which the observer had to decide whether the middle Gaussian blob was shifted upward or downward of the axis defined by the two outer blobs. In one condition, the observers guessed when they were unsure in the normal manner. In another condition, they responded “up” when unsure. Unsurprisingly, the psychometric function was shifted in the latter condition. More interestingly, it was unchanged in slope, in the sense that a fit to the data with one standard deviation and two different means was not significantly better, by a likelihood test, than a fit with two standard deviations and two means. It will be seen that the effect of the guessing rule on the psychometric function was found not only at near-zero cue levels but also propagated out to large values. This is exactly what is predicted from a signal detection model in which the guessing strategy changes the observer’s decision criterion but not the noise.

Morgan et al. also showed that observers could shift the mean of their psychometric functions in a three-dot bisection task, and that they could be trained with feedback to change their criterion without loss of precision. The message of these findings is simple. A shift in the mean of a psychometric function established with MSS could be due to a sensory process like adaptation or attention, but it is logically indistinguishable from a change in the observer’s decisional criterion. The MSS may thus not be the ideal method for the parametric investigation of context effects. The most persuasive context effects, including so-called illusions (Morgan, 1996), are those that can be clearly demonstrated. If a large audience gasps at a demo, this is unlikely to be explained by a subtle change in their decision criterion. The problems arise with effects that are not susceptible to demonstration and where the only evidence we have consists of small effects from the MSS.

### Alternatives to MSS for the measurement of context and illusions

The following section will set out alternatives with examples for measuring illusions without using the MSS. The common theme in these examples is the use of two alternative forced choice (2AFC) with roving pedestals, the aim being to make it hard for the observer to mimic the effects of a genuine perceptual bias with an artificial decision criterion. The merits of the 2AFC (or more generally *m*AFC) procedure as a bias-free measure of sensitivity have been argued many times (Blackwell, 1952; Green & Swets, 1966; Sperling et al., 1990; Jakel & Wichmann, 2006; García-Pérez & Alcalà-Quintana, 2012) and do not need rehearsing here. The use of *m*AFC to measure perceptual biases is less well known. At first sight, the use of a bias-free method to measure a perceptual bias seems to be impossible. Gheorghiu et al. (2011) used spatial 2AFC with two different adapting stimuli in different locations and two different tests in the same locations as the adaptors. The difference between the tests was adjusted keeping their geometric mean constant to find the point at which the two tests seemed the same. However, the observer could still guess in favor of one of the two locations when unsure, and this would be indistinguishable from a perceptual bias induced by the adaptor. This problem can be fixed by using a roving pedestal, as the following examples show. The purpose of the examples is to illustrate the psychophysical methodology, not the details of the stimuli, which have either been published already (Cases 1 and 2) or will be published elsewhere.

## Case 1: Effects of attention on motion adaptation

Some studies have found an effect of attention on adaptation (Chaudhuri, 1990*a*,*b*; Beck et al., 2001; Rezec et al., 2004; Taya et al., 2009) but others have found no effect (Wohlgemuth, 1911; Rees et al., 2001; Morgan, 2011), whereas yet others have found both positive and negative results in conceptual replications (Nishida & Ashida, 2000; Georgiades & Harris, 2002). The problem is that all the positive evidence and most of the negative has come from the MSS or from the duration of the motion aftereffect, which is clearly dependent on a decision criterion. It is hard to decide when a stationary stimulus has stopped moving, and the decision is easily influenced by instructions (Sinha, 1952). A tiny shift in the point of subjective equality (PSE) (much smaller than the JND, Taya et al., 2009) could easily be an expectation effect. What we need is a 2AFC design with interleaved conditions to see if attention does indeed affect adaptation.

Morgan (2013*b*) used an attentional distraction design similar to that in other studies (e.g., Rees et al., 2001). Observers adapted to two spatially separated grating patches moving in opposite directions (L *vs*. R) while carrying out either an easy task or an attention-demanding central task. They then had to decide which of two briefly flashed moving gratings, in the same positions as the adaptors, was moving more quickly. (Note that this is a discrimination of speed, not velocity, since the two gratings could be moving in opposite directions.) One of the two gratings, the standard, always had the same speed. The other, the test, was made slower by an amount that varied over trials in order to determine a psychometric function relating the probability of choosing the reference as a function of the speed difference. The test was presented randomly in the top and bottom positions. In four separate interleaved conditions, the two tests had directions LL, LR, RL, or RR. There is no decision rule such as “report top stimulus as faster,” which can produce consistent results across these four conditions. In fact, however, results were consistent with a single bias corresponding to a reduction in perceived velocity of tests moving in the same direction as their adaptors. There was no effect of attentional load on the value of this bias.

## Case 2: The motion-induced tilt aftereffect

Motion within a stationary aperture causes an apparent shift in position of the aperture in the direction of motion (Ramachandran & Anstis, 1990; De Valois & De Valois, 1991; Hayes, 2000). Thus, two vertically aligned Gabor patches with stationary envelopes and carriers moving in opposite directions appear to have a vernier misalignment. Adaptation to such a stimulus causes a tilt aftereffect in the opposite direction. The existence of a spatiotopic version of this adaptation is controversial (Knapen et al., 2009; Turi & Burr, 2012; Zimmermann et al., 2013). The purpose of an experiment reported by Morgan (2013*a*) was to determine whether this kind of adaptation is retinotopic or spatiotopic, and to confirm a previous report that the strength of adaptation is independent of the relative orientation of adapting and test carrier gratings (McGraw et al., 2002).

Observers adapted to a square array of four Gabor patches with moving carriers that made the square array appear distorted into a trapezoid. They then saw four briefly presented stationary patches in roughly the same position as the adaptors and had to decide whether the left-hand pair or right-hand pair was more tilted from the vertical. The actual angular difference was varied over trials to determine the psychometric function relating choice probability to physical cue. In one condition, the angular cue was in the same direction (+) as that expected from the tilt aftereffect; in the other condition, it was in the opposite direction (−). The two conditions were randomly interleaved. Also, the cue was applied either to the left-hand or the right-hand pair of patches. Since observers do not know on any trial whether they are dealing with a + or a − trial, they cannot mimic a perceptual bias by a guessing rule such as “left when unsure.” The method produced results consistent with a single bias parameter, representing a perceptual shift in the opposite direction to the adaptor. The previous report that the strength of adaptation is independent of the relative orientation of adapting and test carrier gratings (McGraw et al., 2002) was confirmed. In a spatiotopic version of the adaptation, observers moved their eyes between adaptor and test so that the left-hand test fell on the spatial position previously occupied by the right-hand adaptor. Results were consistent with an entirely retinotopic origin of adaptation.

## Case 3: The “rod and frame” effect

**Fig. 2.**
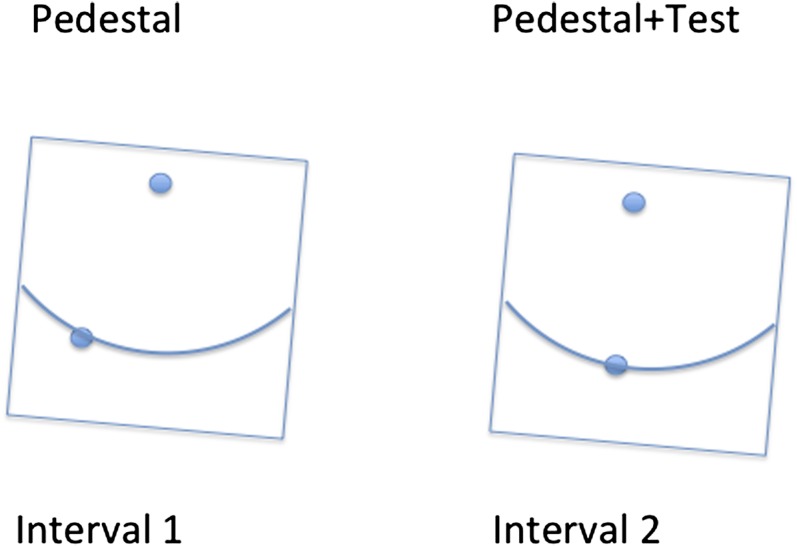
The figure illustrates 2AFC (temporal) task for measuring the effects of frame tilt upon perception of the apparent vertical. The observer’s task is to decide whether the dot pair in the first or second interval is closer to the gravitational vertical. The correct answer in the case illustrated is the “second interval.” The Pedestal was varied between −2, 0, and 2 deg with respect to the true vertical. For further explanation see the text.

A vertical rod seen within a tilted square frame can appear (to some people) tilted in the opposite direction to the frame (e.g., DiLorenzo & Rock, 1982; Dyde & Milner, 2002). We wanted to measure this effect with a 2AFC procedure in as bias-free manner as possible as a preliminary to determining whether the apparent vertical could also be shifted by saccadic adaptation (e.g., Rolfs et al., 2010). First, we wanted to see if the apparent alignment of two separated dots was affected by an outside frame. The MSS method for doing this would present a single dot pair within a frame and ask the subject whether they were tilted clockwise or anticlockwise of the vertical. But this judgment could easily be affected by cognitive factors. All the observer has to do to simulate a frame effect is to adopt the decision rule “when uncertain guess opposite to the tilt of the frame.” The schema for an alternative two AFC (temporal) task not subject to this problem is shown in Fig. 2.

### Methods

Subjects viewed a computer-controlled visual display through a square frame cut out of black card positioned as close to the screen as possible. The room was made as dark as possible by black window blinds. The display screen was a Protouch ATM-173RHOACAD (17″) viewed from 78 cm. The fixation point (white on black background) was placed in the center of a circle (also white) with radius 7.5 deg, visible to the subject as an arc within the frame. The actual position of the fixation point and circle within the frame was jittered over trials to avoid the subject using landmarks on the screen. The rest of the circle was occluded by the frame. Dimensions of the frame were 13.8 × 13.8 deg. In different blocks of trials, the frame was tilted either 0, +7.5, or −7.5 deg.

On each trial, the fixation point was presented for 500 ms, followed by the first 2AFC interval of 1000 ms. During this interval, the fixation point remained visible and a second dot was presented at some point on the circumference of the circle. We define its position by the angle *θ* a line connecting it to fixation would form with the vertical meridian. This was followed after a brief blank interval by the second 2AFC interval, with a dot positioned on the circle at position *θ*′. The subject’s task was to report whether *θ* or *θ*′ was closer to the subjective vertical, in other words “was the first or second dot closer to the vertical with respect to the fixation point?” To clarify what we meant by “the vertical,” we told subjects that the vertical was at right angles to the floor, or equivalently, the shortest line from fixation point to the feet, which were placed directly under the table carrying the monitor. One of the dots, “the standard,” formed a pedestal angle chosen from the list [−2 0 +2] deg with respect to the vertical. The other dot, “the test,” had angle pedestal + *x*, where *x* was chosen from the list [−4 −3 −2 −1 0 1 2 3 4] deg. The temporal order of test and standard was random. Every combination of standard and test was presented eight times in a random order. In other words, the three pedestals were randomly interleaved so the subject could not know on any trial whether either of the dots was in a true vertical position. Nor did they know which was the standard. Thus, no response bias along the lines of “chose the standard if unsure” could masquerade as a perceptual bias. However, with three psychometric functions for the three pedestals, any bias due to frame can be decoded. The experiment was repeated three times with different frame angles [−5 0 5].

## Results

**Fig. 3.**
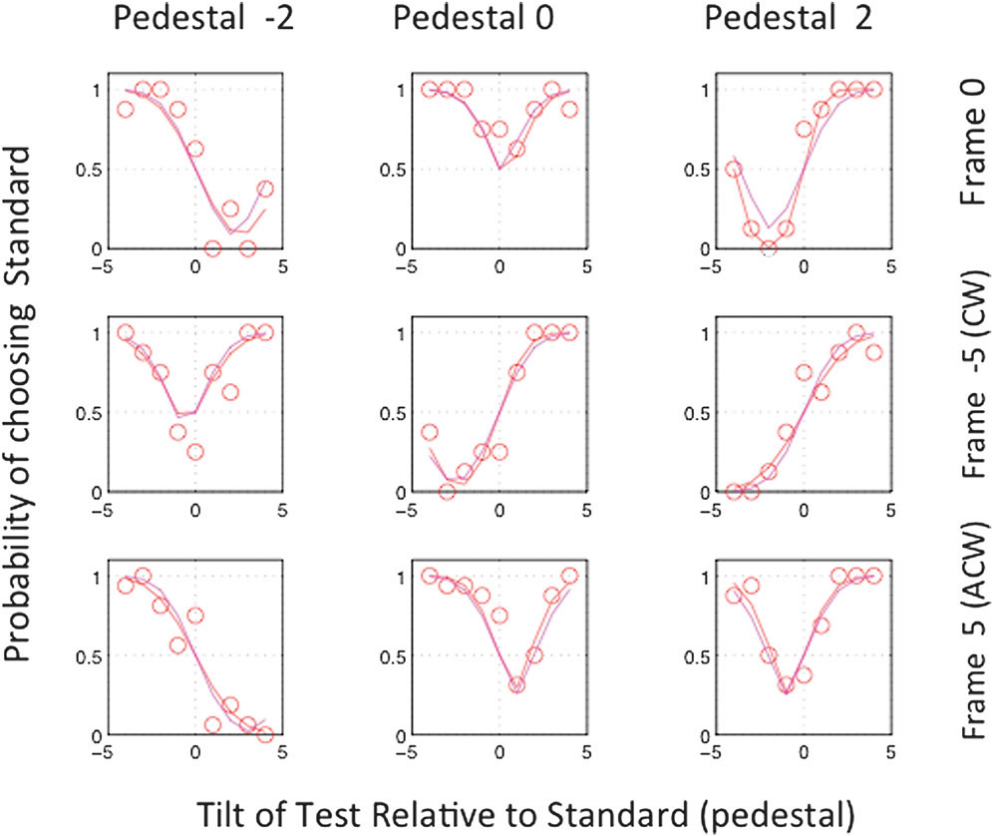
Results for observer MM. Each panel shows the probability of choosing the standard (pedestal) and frame tilt. The nine red curves are individual fits to the data in the nine panels. The magenta curves are four-parameter fits to all the data, assuming constant internal noise and a set of three equivalent pedestals arising from each of the three frames. For further explanation, see the text.

The results for one subject (MM) are illustrated in Fig. 3. Each panel shows the psychometric function for one combination of pedestal and frame. At first sight, the variety of psychometric functions is bewildering, but it is actually easy to decode. The simplest case is 0 pedestal and 0 frame (top row, second column of the figure). Here, the pedestal is always vertical and is seen as such. The angular cue applied to the test moves it away from the vertical in either direction, resulting in increased probability of choosing the standard as being more vertical. Now consider pedestal −2 and frame 0. Positive angles to the test bring it closer to the vertical than the standard, resulting choice probabilities for the standard of *P* < 0.5, and so on. The red curves are fits of the function *p* = *Φ*([|ped + test + *μ*(*f*)| − |ped + *μ*(*f*)|]/*σ*(*f*)), where *p* is the probability of choosing the pedestal, *Φ* is the normal c.d.f., and *μ*(*f*) and *σ*(*f*) are the frame-specific bias and frame-specific internal noise, respectively. The magenta curves are fits of the function *p* = *Φ*([|ped + test + *μ*(*f*)| − |ped + *μ*(*f*)|]/*σ*), where *σ* is the same in all frame conditions.

**Fig. 4.**
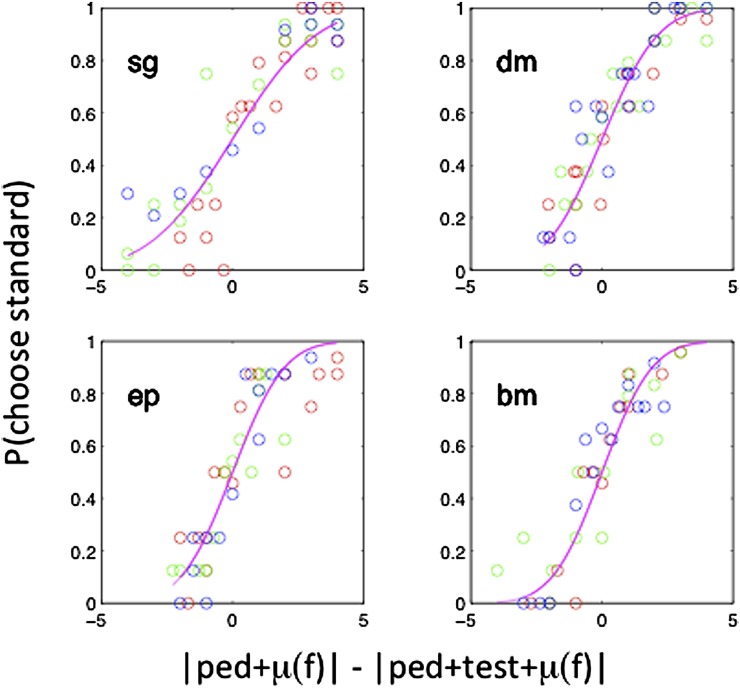
The figure replots data of the kind previously shown in Fig. 3, with different symbols for the three frame conditions (red, frame 0, green: frame CW, and blue, frame ACW). Each panel shows the results for a single subject. The fitted magenta curve assumes different values of *μ*(*f*) for the three frame conditions but the same value of internal noise, *σ*. *p* = Φ([|ped + test + *μ*(*f*)| − |ped + *μ*(*f*)|]/*σ*).

Since the nine “magenta” functions in Fig. 3 differ in only one free parameter *μ*(*f*), they can be collapsed into three functions for the three different frames. This is done in Fig. 4 for each of the remaining four subjects in the experiment. The three frame conditions are indicated by different colors.

Table 1 shows the values of the frame bias *μ* and internal noise for each of five subjects tested so far, two of them (EP and BM) being entirely naïve. To interpret the sign of *μ* recall that we expect a CW frame to cause an apparent ACW tilt of the virtual line joining the fixation point to the target dot equivalent to a positive angular shift. Thus, *μ* for a CW frame should be positive. Three subjects (MM, SG, and EP) showed an effect of the frame in the direction of the classical “rod and frame” effect. The other two (DM and EP) showed no clear effect. Individual differences in the rod and frame are well established (e.g., Spinelli et al., 1995), and we are currently carrying out head-tilt experiments to determine whether they arise from different use of gravitational *versus* visual cues.

**Table 1.** Fitted values for biases (μ) and internal noise (σ) in the Rod and Frame Experiment (Case 3)

Subject	*μ* 0 tilt	*μ* CW frame	*μ* ACW frame	*σ*
MM	0.146	2.56	−1.24	1.54
SG	0.17	6.02	−11.98	2.51
DM	0.52	0.28	−0.61	1,68
EP	0.35	−0.64	−0.24	1.54
BM	−0.8	2.45	−1.18	1.49

The method for determining the frame effect relies upon comparison with an internal standard, in this case of the vertical. For many other context effects, like the Muller-Lyer effect, there is no such standard, so it must be provided as an external reference. The next case describes an example.

## Case 4: The Ebbinghaus circles surround effect

**Fig. 5.**
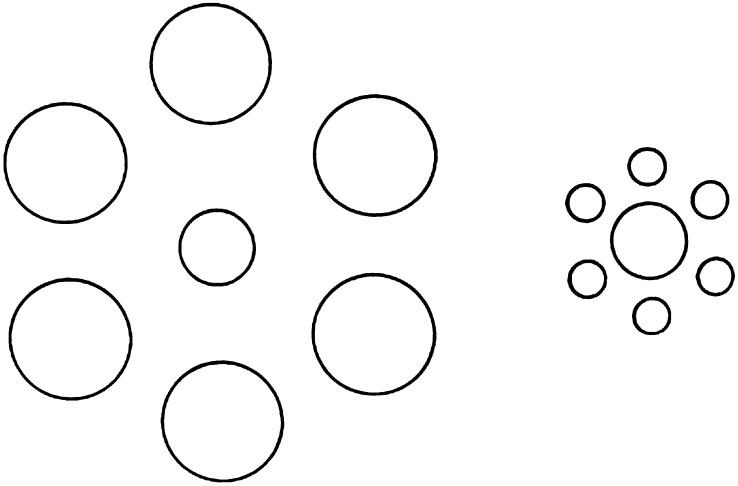
The Ebbinghaus size context effect. The central circle on the left may appear smaller than the equal size circle on the right.

The Ebbinghaus size context effect (Fig. 5) has become topical because of (disputed) claims that it does not affect grip aperture when subjects try to pick up real three objects (Goodale & Milner, 1992; Franz, 2001; Franz et al., 2001; Glover & Dixon, 2002).

### Methods

Stimuli were presented on the LCD display of a MacBookPro laptop computer with screen dimensions 33 × 20.7 cm (1440 × 900 pixels) viewed at 0.57 m so that 1 pixel subtended 1.25 arcmin visual angle. The background screen luminance was 50 cd/m^2^.

**Fig. 6.**
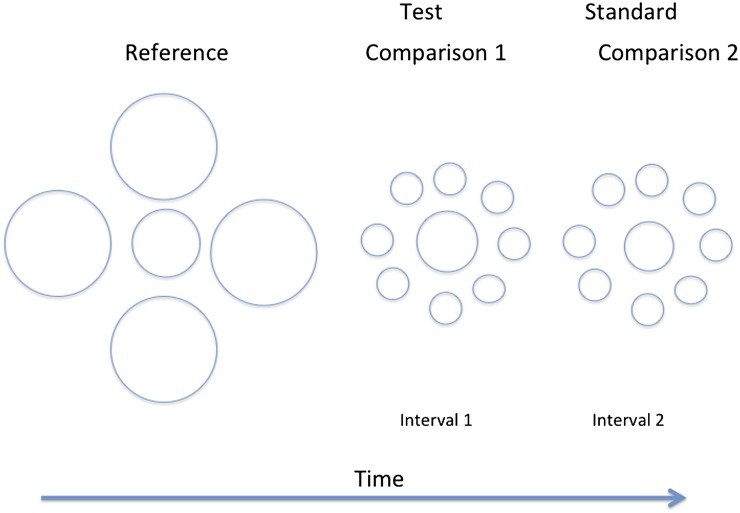
Schema of the stimulus sequence for measuring the Ebbinghaus context effect with a two AFC method. First, the reference circle and the context were shown, which did not vary in physical size throughout the experiment. Then the two comparison stimuli were presented in sequence. One of these was the standard, which had a pedestal size difference relative to the reference. The other was the test, which was varied in size relative to the standard over trials in order to determine a psychometric function. The subject’s task was to decide whether the first or second comparison stimulus was nearer in size (radius) to the reference. For further details, see the text. The condition illustrated is called L/S (large surround reference/small surround comparison) in the text.

In the case of size discrimination, the 2AFC method cannot use an implicit standard such as the vertical, so a reference must be provided. This was done using the temporal sequence illustrated in Fig. 6. First, the “reference” circle was shown (1 s) at a physical size that did not vary throughout the experiment, although its context did. Then the two “comparison” stimuli were presented in sequence (0.5 s each). One of these was the standard, which had a pedestal size difference relative to the reference. The other was the test, which was increased in size relative to the standard over trials in order to determine a psychometric function. The subject’s task was to decide whether the first or the second comparison stimulus was nearer in size (radius) to the standard. Standard and test were presented in a random order. Three different pedestals were randomly interleaved: −5, 0, and +5% (relative to the reference). Three context conditions were used corresponding to the three frame conditions in the previous experiment. In the control case, both the reference and comparison stimuli had a small circle context (Condition S/S). In Condition S/L, the reference had a small circle context and the comparison stimuli both had a large circle context. Condition L/S was the reverse of S/L. Note that the context for standard and test (the comparison stimuli) was always the same. The radius of the reference circle was 37.5 arcmin. The S context comprised eight circles each of radius 12.5 arcmin separated (center to center) from the center of the array by 12.5 arcmin. The L context comprised four circles each of radius 50 arcmin separated (center to center) from the center of the array by 125 arcmin. The relative dimensions were closely similar to those in Fig. 6.

Data were obtained for two subjects (the author and a graduate student NN).

**Fig. 7.**
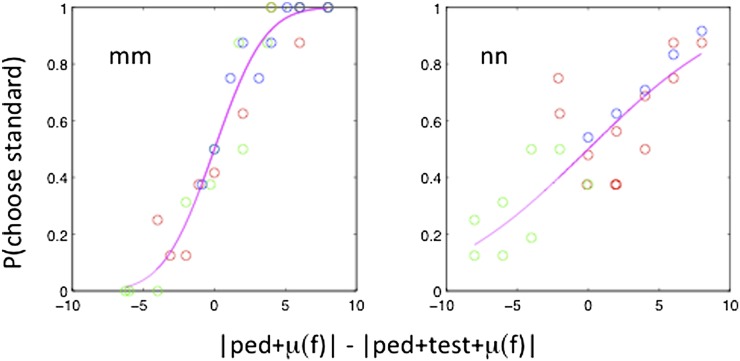
Figure shows psychometric functions taking into account the pedestal value and fitted context bias for each observation. The *x*-axis represents the internal signal on which the observer is assumed to base his decision. The different contexts are color coded: Condition S/S (red), Condition S/L (green), and Condition L/S (blue).

The logic of the experiment closely followed that of the previous experiment (Case 3, the frame effect) so results are presented (Fig. 7) in the same way in Fig. 4. Once again, the effect of the context was modeled as an equivalent pedestal, with no effect on internal noise.

The results are summarized in Table 2. The two observers had biases in the same direction, but those of NN were greater.

**Table 2.** Fitted values for biases (μ) and internal noise (σ) in the Ebbinghaus Circles Experiment (Case 4). The units are Weber Fractions of circle radius (100*Δr/r)where Dr is the difference in radius between the two comparison stimuli

Subject	*μ* context S/S	*μ* context S/L	*μ* context L/S	*σ*
MM	0.49	−2.136	3.56	2.89
NN	0.20	−13.84	12.25	8.10

## Case 5: The Poggendorff effect

The “Poggendorff effect” is a classical geometric illusion in which actually collinear lines appear noncollinear, probably at least in part because of orientation repulsion (see Morgan, 1999 for review and evidence from MSS that the effect is increased by blur and is second order in terms of contrast). The experiment described here used the vertical version of the Poggendorff with two vertical parallels and two 45 deg oblique pointers.

### Methods

As in the previous experiment, stimuli were presented on the LCD display of a MacBookPro laptop computer with screen dimensions 33 × 20.7 cm (1440 × 900 pixels) viewed at 0.57 m so that 1 pixel subtended 1.25 arcmin visual angle. The background screen luminance was 50 cd/m^2^.

The procedure was 2AFC, temporal. One interval contained a standard with one of three randomly interleaved pedestal values of misalignment relative to the true alignment point. The other contained a test with an upward displacement relative to the standard sampled from four prearranged levels. Test and standard were presented in a random temporal order. Observers decided which interval contained the more aligned stimulus. Note that this could be the test or the standard depending on the pedestal and the size of the putative Poggendorff bias. The observer has no way of knowing which interval is the test, which the standard, or which is really more aligned. The psychometric functions for the three pedestal levels were analyzed together with a two parameter (*σ*; *μ*) model as in the two previous cases. The subjects were the three authors.

**Fig. 8.**
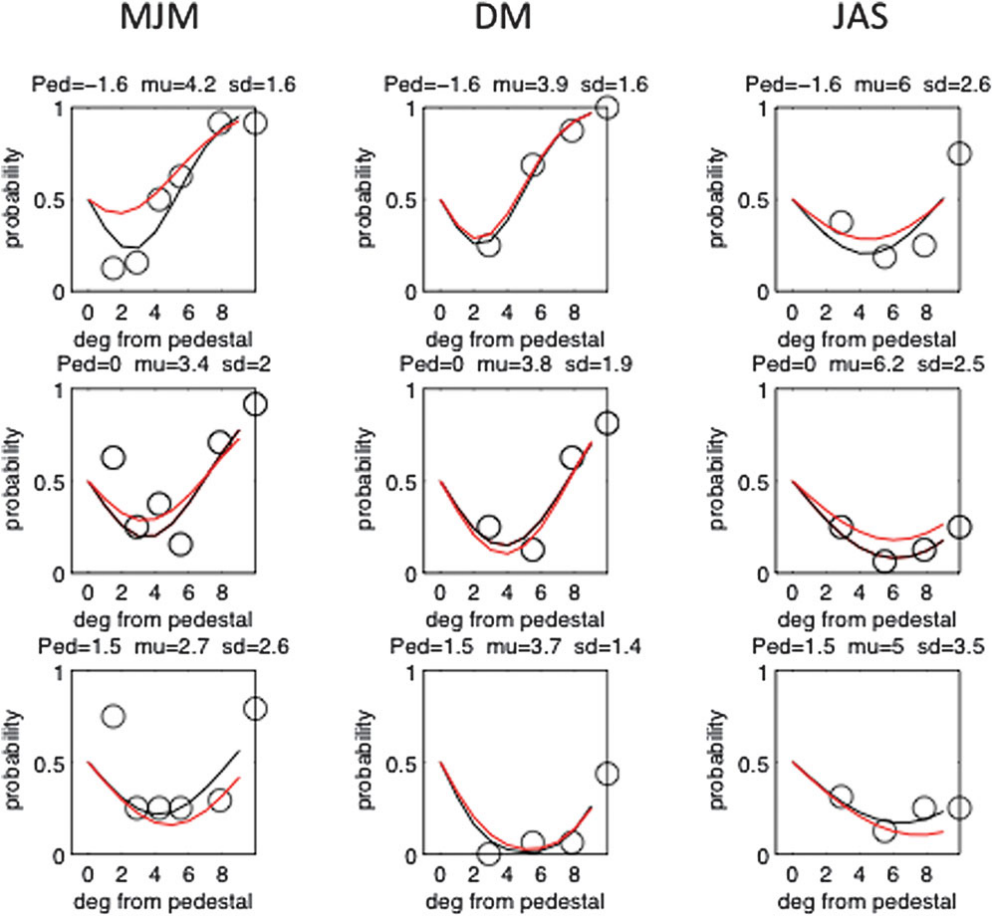
Each panel shows the probability of choosing the standard (pedestal) as being more collinear with the pointer than the test at each of three pedestal levels (rows) in three different subjects (columns). The black curves are individual two parameter fits to the data in the nine panels. The red curves are two-parameter fits to the data combined over pedestals, assuming constant internal noise. For further explanation, see the text.

Bias was defined as in previous examples as the equivalent of an additional pedestal. In this case, the bias was converted from displacement units to degrees of rotation, that is, the misalignment that would be produced by rotating the pointer around its intersection with the vertical inducer. The data (Fig. 8) revealed a bias in the direction of the Poggendorff effect ranging from 2.7 to 6 deg. The biases in a two-parameter (*μ*; *σ*) fit when all three pedestals were combined were: 3.4 deg (MJM), 4.15 deg (DM), and 3.9 deg (JAS). Following these measurements, the bias was also determined by the MSS, using the conventional method of guessing whether the right-hand pointer was higher or lower than the imaginary point of collinearity with the right-hand pointer. These values were: 0.67 deg (MJM), 2.86 (DM), and 1.75 (JAS). We cannot say why these values were lower in every case than the 2AFC values, but one possibility (apart from an order effect) is that experienced subjects tend to equalize the two response alternatives.

### Other cases

It is left to the reader as an exercise to design roving pedestal 2AFC methods for measuring the Muller-Lyer illusion and other effects of context such as contrast–contrast (Chubb et al., 1989).

## Conclusion

Class A/Type 1 psychophysical procedures occupy a privileged position because they are based on a clear linking hypothesis and make no reference to subjective qualities of the stimulus. They can be performed on machines to measure their intrinsic noise on animals or, indeed, in human subjects who are not conscious of the stimuli. The purpose of Class B/Type 2 procedures is to measure bias; therefore, they can never be bias free. However, there are at least three sources of bias, which should be the goal of a correctly designed experiment to separate: (1) response biases, (2) decisional biases such as deciding in favor of one alternative when unsure, and (3) perceptual bias. The problem with MSS is that it confounds all three. We have argued that 2AFC can do a better job in separating decisional and perceptual biases particularly if a roving pedestal is used. The growing and worrying number of contradictions appearing in the literature using MSS could possibly be stemmed by using *m*AFC instead.

## Footnotes

1 The problem, as argued in Morgan (2003), is to show that other neurons are unnecessary. We cannot deprive a neuron of its input and output and determine that it has sensations. The idea that a single neuron can have a sensation is tantamount to “the secretion theory” Huxley attributed to Cabanis.

2 We could use feedback and see if this flattens the psychometric function during early trials while the observer establishes a new criterion. Potentially, this is a powerful Class A measurement of a genuine perceptual bias and could be used, for example, to test the claim that autistic children are supernormal at ignoring context.
